# Assessing immune infiltration and the tumor microenvironment for the diagnosis and prognosis of sarcoma

**DOI:** 10.1186/s12935-020-01672-3

**Published:** 2020-12-02

**Authors:** Naiqiang Zhu, Jingyi Hou

**Affiliations:** Department of Minimally Invasive Spinal Surgery, Affiliated Hospital of Chengde Medical College, Chengde, 067000 China

**Keywords:** Sarcomas, Immune infiltration, Prognosis, Weighted gene co-expression analysis, Tumor microenvironment

## Abstract

**Background:**

Sarcomas, cancers originating from mesenchymal cells, are comprehensive tumors with poor prognoses, yet their tumorigenic mechanisms are largely unknown. In this study, we characterize infiltrating immune cells and analyze immune scores to identify the molecular mechanism of immunologic response to sarcomas.

**Method:**

The “CIBERSORT” algorithm was used to calculate the amount of L22 immune cell infiltration in sarcomas. Then, the “ESTIMATE” algorithm was used to assess the “Estimate,” “Immune,” and “Stromal” scores. Weighted gene co-expression network analysis (WGCNA) was utilized to identify the significant module related to the immune therapeutic target. Gene ontology (GO) enrichment and Kyoto Encyclopedia of Genes and Genomes (KEGG) analyses were performed using the “clusterProfiler” package in R for annotation and visualization.

**Results:**

Macrophages were the most common immune cells infiltrating sarcomas. The number of CD8 T cells was negatively associated with that of M0 and M2 macrophages, and positively associated with M macrophages in sarcomas samples. The clinical parameters (disease type, gender) significantly increased with higher Estimate, Immune, and Stromal scores, and with a better prognosis. The blue module was significantly associated with CD8 T cells. Functional enrichment analysis showed that the blue module was mainly involved in chemokine signaling and the PI3K-Akt signaling pathway. *CD48, P2RY10* and *RASAL3* were identified and validated at the protein level.

**Conclusion:**

Based on the immune cell infiltration and immune microenvironment, three key genes were identified, thus presenting novel molecular mechanisms of sarcoma metastasis.

## Background

Sarcomas are a widespread, heterogeneous group of tumors that occur on the skin, under the skin, in the periosteum, and on the ends of the long bones of adolescents and the elderly (overall incidence: 1–2/100,000 annually) [1], which is characteristic of cancers originating from mesenchymal cells [2]. Histopathologically, sarcomas are classified as either bone or soft tissue sarcomas [3]. To date, the etiology of sarcomas is not well characterized; however, their incidence appears to be associated with heredity [4], viral infection [5], trauma [6], environmental factors [7], and exposure to radiation [8]. Compared with other cancers, the degree of malignancy of sarcomas is relatively high [9], and hematogenous metastasis can spread to various organs, such as lung, brain, liver, and bone [10, 11]. Since sarcomas rarely display clinical manifestations in the initial stages of the disease, most sarcomas are diagnosed at more advanced stages.

Standard of care for patients with sarcoma is mainly comprised of local surgery, chemotherapy, and radiotherapy [12]. Among these, radical surgery or amputation is the most common [13]. Chemotherapy and radiotherapy are also included before and after surgery to prevent recurrence [14]; however, the success rate of treatment is currently low, and many patients still have poor prognoses and die of cancer-related causes. For some patients with distant metastases, palliative local treatments are chosen to control and delay disease progression [15]. With the enhanced study of the immune system, immunotherapy has emerged as very promising method to treat sarcomas after surgery and chemotherapy [16]. Researchers have adopted various immunotherapy strategies for different types of immune deficiency, but the main treatment obstacles are identifying the specific target antigen and dealing with the severe adverse effects of the selected treatment [17]. Additional research is urgently needed to identify ways to ameliorate the toxic response to immunotherapy, identify specific targets related to sarcoma, improve the effectiveness and safety of immunotherapy, and design new combinations of immune checkpoint inhibitors and other therapies [18]. Therefore, the characterization of sarcoma-specific biomarkers and the molecular mechanisms responsible for the transformation of normal cells to sarcoma are essential for the success of sarcoma immunotherapy.

Since immune infiltration and the tumor microenvironment may predict potential molecular mechanisms of sarcomas, we used the CIBERSORT (Cell-Type Identification by Estimating Relative Subsets of RNA Transcripts) algorithm to characterize differential expression patterns of immune cell infiltration between sarcoma samples and normal samples in 22 subpopulations of immune cells, and we used the “Estimate” algorithm to analyze the Stromal and Immune scores of differential gene expression. Then, we used weighted gene co-expression network analysis (WGCNA, https://bmcbioinformatics.biomedcentral.com) to identify several key genes related to immune therapeutic target, to suggest novel molecular mechanisms responsible for the transformation and growth of sarcomas.

## Materials and methods

### Sample acquisition and prepossessing

The sarcoma transcriptome was downloaded from the TCGA database [19] via the Genomic Data Commons (GDC) data portal. Clinical information of sarcoma patients was also acquired from the TCGA. Filter criteria for eligible samples were as follows: (1) samples with both transcriptome data and clinical information were included; (2) samples with duplicated data or null values were excluded.

### CIBERSORT evaluation

The “limma” package in R was used to normalize the data to estimate the percentage of infiltrating immune cells, and then standardized gene expression data were uploaded to CIBERSORT [20, 21]. Of these, the LM22 signature and 1,000 permutation were added [22], with CIBERSORT cases (*p* < 0.05) included in the survival analysis.

### Estimation evaluation

Estimate-, Immune- and Stromal scores of sarcoma samples were calculated with the ESTIMATE algorithm of the “estimate” package [23]. The “limma” package in R [24] was applied to identify differentially expressed genes (DEGs) with *p*-values < 0.05, and |log fold change (FC)|> 1. Then, the relationship between DEGs and overall survival of the patients from whom the sarcoma samples were acquired was analyzed with the “survival” package in R [25].

### Co-expression analysis

Samples identified by both CIBERSORT and ESTIMATE were included in the co-expression analysis. The co-expression analysis was performed using the WGCNA package in R language [26, 27]. To maintain the gene network level off to the scale-free topology and enough connectivity, four were identified as the best soft power threshold when the degree of independence was 0.8. Then, gene modules were detected based on a topological matrix (TOM). Genes with high correlation were divided into one module (minimum module size = 20). To further evaluate the robust and reliability of the modules, the permutation test (50x) was performed with the “modulePreservation function.” [21]. As instructed, the modules with a Zsummary.qual < 5 were not considered stable in the co-expressed network. Modules with Zsummary < 2 indicated “no preservation,” 2 < Zsummary < 10 indicated “weak preservation,” and Zsummary > 10 indicated “strong preservation” (which is recommended to be used as the significant module). Modules with a Zsummary < 10, and a Zsummary.qual < 5 were excluded from the following analysis [28, 29].

### Identification of significant modules and functional analysis

In this study, gene significance (GS) [30] was used to calculate the correlation coefficients. For example, the module significance (MS) was indexed as the average GS for the genes in given module. When the modules with the highest MS values were regarded as the significant modules. The analysis of genes in significant modules was performed using gene ontology (GO) enrichment and Kyoto Encyclopedia of Genes and Genomes (KEGG) pathway analysis, included in the “clusterProfiler” package [31] in R, with *p*-values < 0.05.

### Gene identification and validation

Module connectivity (cor.geneModule Membership [MM]) and clinical trait relationship (cor.geneTraitSignificance) of genes in the significant module were calculated. In addition, the genes identified as significant were uploaded to the STRING database (http://string-db.org) (confidence > 0.1) [32], and Cytoscape was performed to establish the protein–protein interaction (PPI) network. The MCODE plug-in was utilized to identify the most significant sub-module, with a degree cut-off of 2, k-score of 2, and max. depth of 100. Then, we used the “Network Analyzer”to identify nodes in the network, in which the size and the color of the nodes represent MCODE scores. Genes with cor.geneModule > 0.8, cor.geneTraitSignificance > 0.2, and the largest MCODE sub-network were considered for further analysis. For further validation, identified genes were analyzed with the “survival” package in R, where *p* < 0.05 was considered statistically significant. In addition, the Human Protein Atlas Database (HPAD) [33] was used to validate the protein -level of these genes.

### Genetic alterations

CBio Cancer Genomics Portal [34, 35] is an open-access website used for the visualization and analysis of various cancers. In this study, this platform was utilized to investigate and compare the genetic alternations of the hub genes.

## Results

### Workflow analysis and data description

**Fig. 1:**
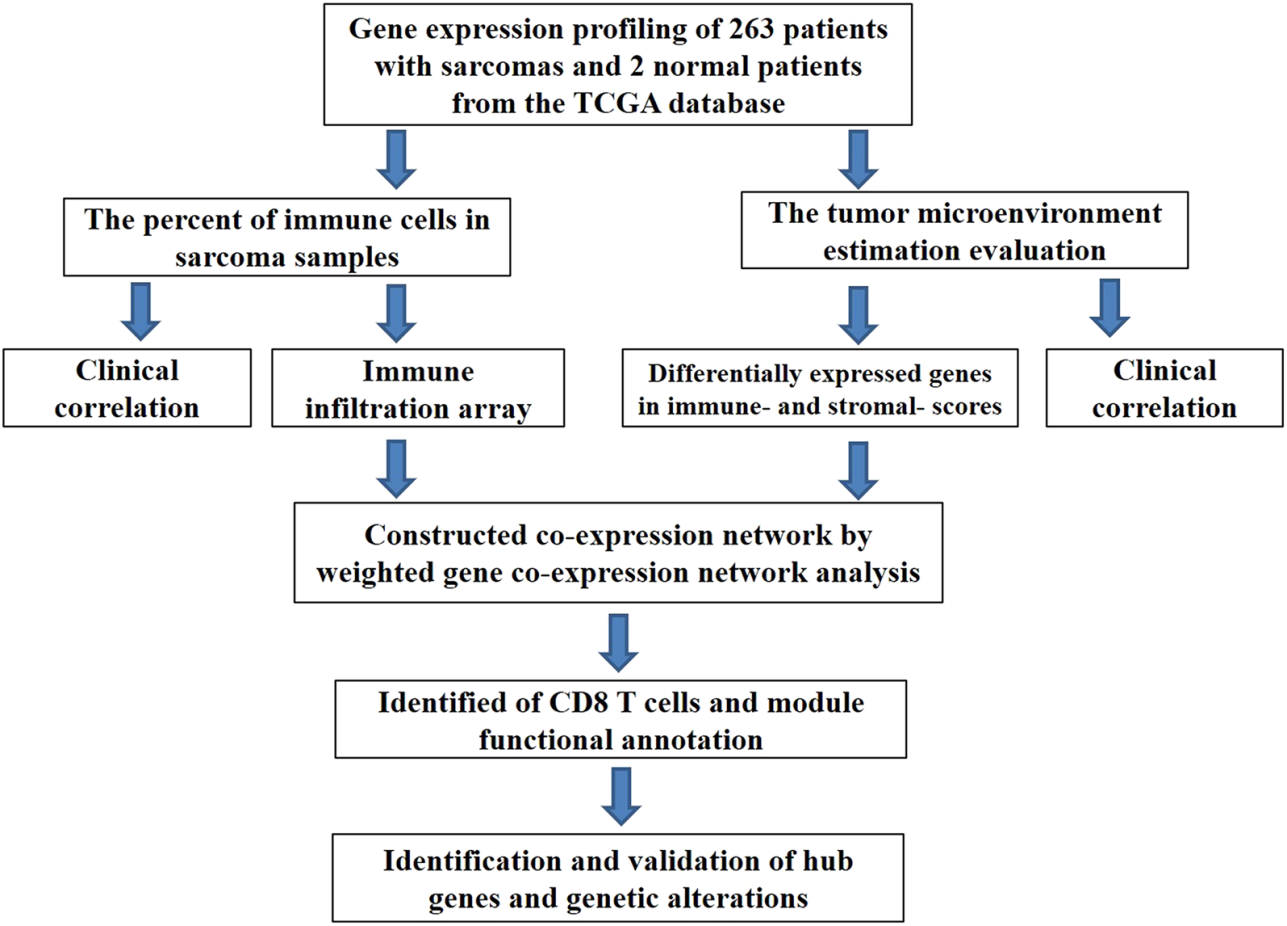
Flow chart of the analytical process

The analytical workflow is shown in Fig. 1. First, we evaluated the differences in immune cell infiltration and tumor microenvironments of sarcoma tissues and normal, adjacent tissues. Next, we characterized the significant module associated with CD8 T cells by WGCNA, and hub genes were identified and validated. Samples from 263 patients with sarcoma and two samples of normal, adjacent tissues were obtained from the TCGA database for further analysis.

### The percent of tumor-infiltrating lymphocytes in sarcoma samples and clinical correlation

**Fig. 2:**
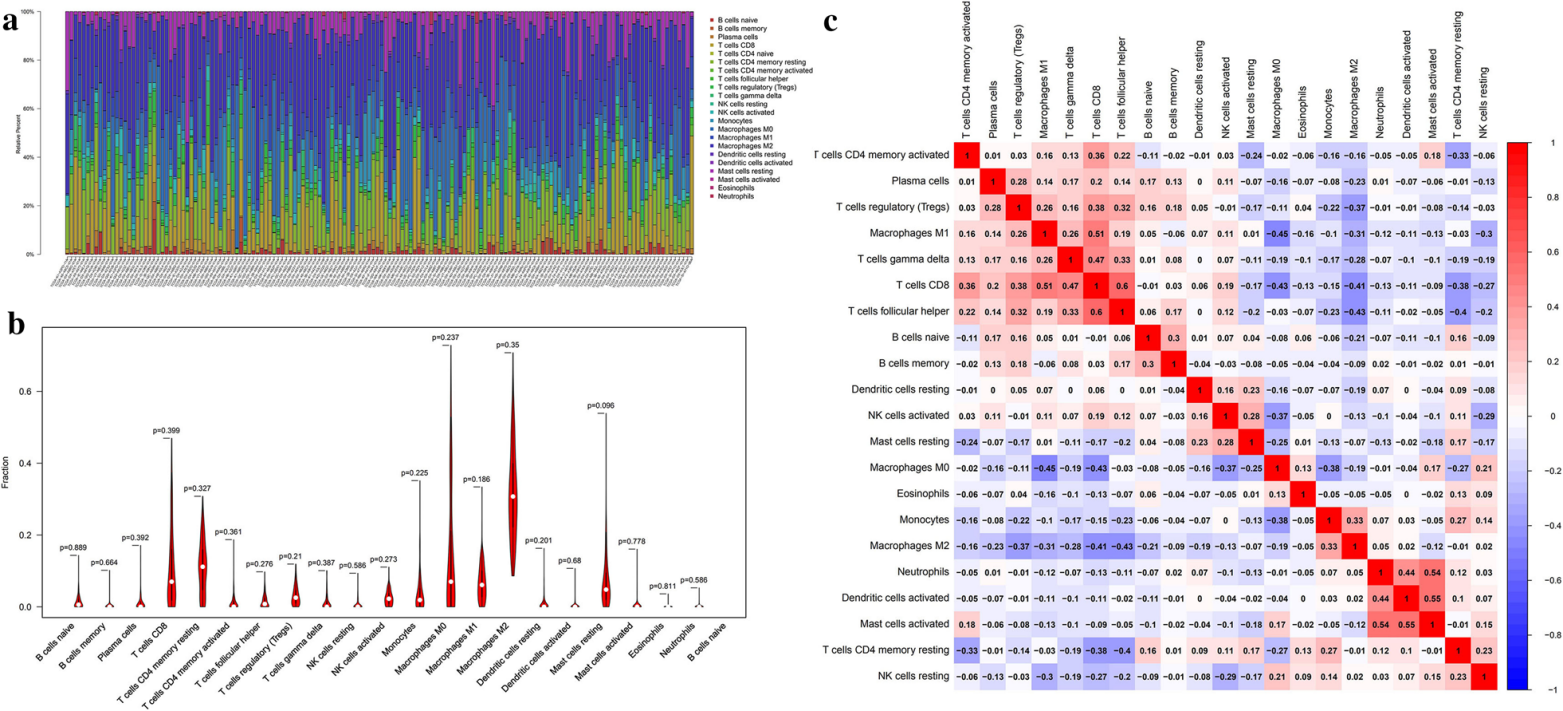
**a** Composition of immune cells. **b** Violin plot of immune cells. **c** Co-expression patterns among fractions of immune cells

**Fig. 3:**
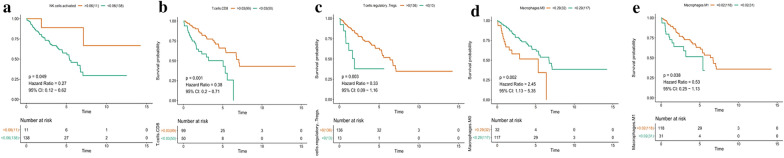
Survival curves of the fraction of **a** activated NK cells, **b** CD8 T cells, **c** regulatory T cells, **d** M0 macrophages, and **e** M1 macrophages

CIBERSORT is an algorithm, based on the machine-learning, highly sensitive and specific discrimination of 22 human immune cell phenotypes in several cancer types [36, 37]. To estimate the immune cell composition, the CIBERSORT was used to quantify the relative levels of distinct tumor-infiltrating lymphocytes (TILs). In this study, we used CIBERSORT algorithm to assess the composition of immune cells in sarcoma and normal samples (Fig. 2a, b, Additional file 1: Figure S1). Among these TILs, the macrophages were the predominant immune cell type in sarcoma tissues. The fraction of CD8 T cells was negatively associated with M0 macrophages (R = −0.43) and M2 macrophages (R = −0.41), and positively associated with M1 macrophages (R = 0.51) and follicular, helper T cells (R = 0.6) (Fig. 2c). we performed a Kaplan–Meier survival analysis to evaluate the correlation between the 22 immune cell subtypes and the prognosis of patients from whom the tumors were acquired. The fraction of activated dendritic cells (p = 9.293e−04), resting dendritic cells (p = 0.031), M2 macrophages (p = 1.668e−04), neutrophils (p = 0.046), and plasma cells (p = 0.031) were significantly different among disease types. The fractions of activated dendritic cells (p = 0.04) and M2 macrophages (p = 0.023) were significantly different among disease recurrence, and the fraction of activated dendritic cells (p = 0.021), resting NK cells (p = 0.007), and follicular helper T cells (p = 0.028) were also significantly different among total necrosis percent (Additional file 2: Figure S2). In addition, the fractions of activated NK cells (p = 0.049), CD8 T cells (p < 0.001), regulatory T cells (p = 0.003), M0 macrophages (p = 0.002), and M1 macrophages (p = 0.038) were significantly correlated with overall survival (Fig. 3).

### Immune-, stroma- and estimate- scores correlated with clinical parameters

**Fig. 4:**
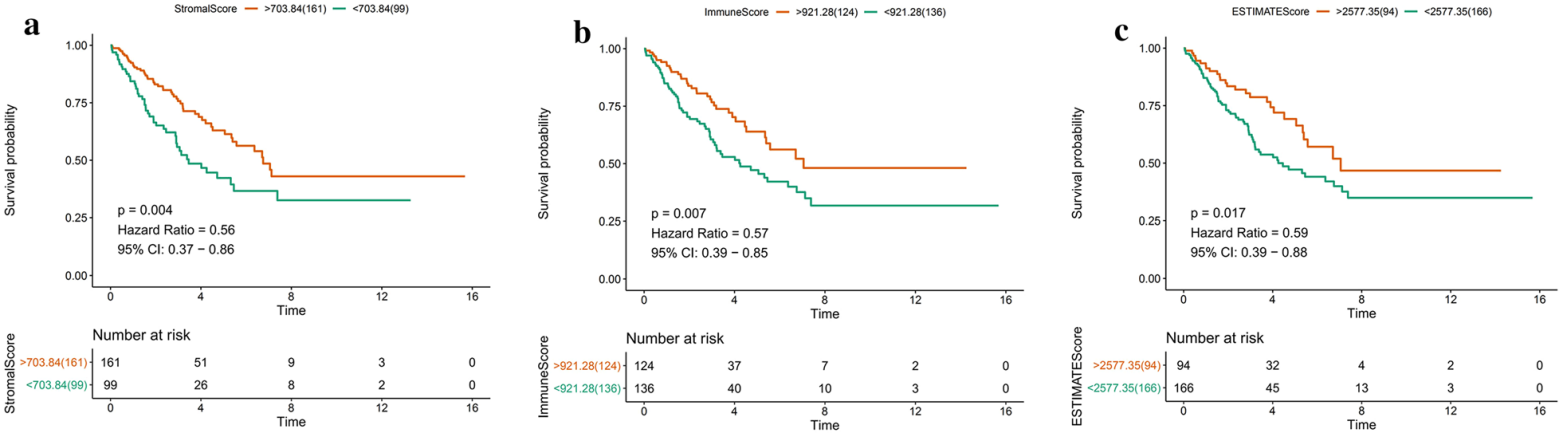
The association between **a** Estimate-, **b** Immune-, **c** Stromal scores of sarcoma tumors and the overall survival of patients harboring these tumors

To evaluate the microenvironment infiltration of immune and stromal, we applied the “ESTIMATE” package in R to match and calculate the Immune-, Stromal-, and Estimate- scores of 263 patients. As shown in Table 1, the stromal scores ranged from −1336.63 to 2476.22, the Immune scores ranged from −1722.08 to 3499.2, and the Estimate scores ranged from −2977.43 to 5336.41. The “survival” package in R was utilized to analyze the correlations of Estimate, Immune, and Stromal scores with overall survival (Fig. 4). The patients with tumors that had high Estimate, Immune, and Stromal scores had a significantly better prognosis than patients with tumors that had low Estimate, Immune, and Stromal score group (p = 0.004, p = 0.007, p = 0.017, respectively). Furthermore, the relationship among Estimate-, Immune-, Stromal- scores and clinical parameters were evaluated (Additional file 3: Figure S3). The clinical parameters disease type, gender significantly increased as Estimate-, Immune-, and Stromal- scores increased (*p* < 0.05).

**Table 1:** Estimate scores, Immune scores, Stromal scores, and clinical parameters of sarcoma samples

Characteristic	N	Estimate score (range)	Immune score (range)	Stromal score (range)
Age				
< 61.5	135	−2977.42 to 5336.40	−1722.08 to 3499.29	−1336.63 to 2476.22
≥ 61.5	128	−2142.73 to 5178.61	−1135.92 to 3382.39	−2142.73 to 5178.61
Gender				
Female	144	−2886.10 to 5336.41	−1722.09 to 3499.29	−1336.63 to 2032.58
Male	119	−2977.43 to 4985.82	−1646.75 to 3382.38	−1330.67 to 2476.22
Disease type				
Fibromatous	40	−504.94 to 5336.41	−962.99 to 3499.29	153.81 to 2157.00
Lipomatous	60	−1889.11 to 5187.61	−1110.77 to 3382.39	−778.34 to 2476.22
Myomatous	106	−2114.77 to 4221.40	−1520.77 to 2998.72	−727.47 to 1727.86
Nerve sheath	10	−490.23 to 3520.43	−733.46 to 2271.22	−122.82 to 1249.22
Soft tissue	37	−274.38 to 4985.82	−496.70 to 3348.72	106.11 to 1731.42
Synovial-like	10	−2977.43 to 726.05	−1722.08 to −174.09	−1336.63 to 900.13
Tumor total necrosis (%)				
0%	71	−2886.10 to 5336.41	−1722.08 to 3499.29	−1336.63 to 2230.57
< 10%	38	−1645.38 to 4600.14	−1067.28 to 3027.08	−628.27 to 1985.01
≥ 10, < 50%	62	−2977.43 to 4510.53	−1646.76 to 3140.17	−1330.67 to 1793.80
> 50%	12	−2114.77 to 3725.37	−1520.77 to 2293.27	−727.46 to 1707.18
Unknown	80	−1889.11 to 5178.61	−1110.77 to 3199.34	−778.34 to 2476.22
Disease recurrence				
Yes	29	−2647.50 to 4104.67	−1722.08 to 2386.97	−925.41 to 1985.01
No	146	−2977.43 to 5336.41	−1646.76 to 3499.29	−1336.63 to 2230.57
Unknown	88	−1889.11 to 5178.61	−1110.77 to 3199.35	−778.34 to 2476.22

### Identification of DEGs and clinical correlation

To further investigate the function of pivotal gene in microenvironment infiltration, the gene expression profiles were differentiated into two groups via differentially genes analysis (high vs. low, Additional file 4: Figure S4). Using the Immune scores, a total of 2199 DEGs between the high-score and low-score group were identified in which 1070 genes were upregulated, and 1129 genes were downregulated. Similarly, for stromal scores, 995 DEGs were upregulated and 1498 DEGs were downregulated between the high-score and low-score groups. The genes differentially upregulated and downregulated in the high vs. low immune and stromal scores groups were further analyzed. Of these 1509 genes, 729 were upregulated and 780 were downregulated (Additional file 5: Figure S5).

### Weighted gene co-expression network construction and module preservation analysis

WGCNA focused on transforming gene expression data into co-expressed module and screening out hub genes, providing insights into correlation between genes in different samples [26]. After validation, the 1509 DEGs were used to create a co-expression network using WGCNA. The analysis was performed as previously described [27]: Briefly, the gene expression profile matrix of all samples was transformed into a Pearson’s correlation coefficient matrix, and the distribution of connections among these genes indicated that the profile met the criteria of a scale-free network. Then we constructed network based on the scale-free network (Additional file 6: Figure S6A). We assigned a power value of 4 with a 0.8 degree of independence (Additional files 6, 7: Figure S6, S7). The size of the seven modules ranged from 21 to 338 genes. Genes that were not co-expressed were assigned to the grey module, and were not further analyzed. Furthermore, the results of module stability analysis demonstrated that the Zsummary.qual for module preservation of gold modules was < 5, and the Zsummary statistic for module preservation of the blue, green, yellow, turquoise, and brown modules was > 10 (Additional file 6 Fig. 6c, Additional file 8: Table S1). Based on these data, the gold module was not considered stable in these analyses, and it was not used in subsequent analyses.

### Identifying significant modules and module functional annotation

The association of the six modules with types of immune cell infiltration was analyzed (Additional file 9: Figure S8), and the blue module was identified as having the highest correlation with CD8 T cells compared with other modules (cor = 0.9, p = 7.7e−63). The eigengenes and adjacencies were calculated according to their correlation (Additional file 9: Figure S8), and the five modules were divided into two main clusters. Furthermore, a heatmap was produced based on the interaction relationship of the six modules (Additional file 9: Figure S8). CD8 T cells recognize and kill cancer cells by expressing cytokines and cytotoxic molecules and, thus have been identified as a key target for immunotherapy. The blue module had the highest correlation with CD8 T cells, which suggested that the genes in blue module were candidates for immunotherapy biomarkers of sarcoma (Additional files 9, 10: Figure S8, S9).

To further elucidate the function of the significant module, all genes in the blue module were analyzed with the “clusterProfiler” package in R to identify representative KEGG pathways and GO terms. As shown in Additional file 11: Figure S10, the most significantly enriched pathways of the blue module following KEGG pathway analysis were enhanced in chemokine signaling, the PI3K-AKT signaling pathway, and the JAK-STAT signaling pathway (Additional file 12: Table S2). GO enrichment analysis showed that the blue module contained biological processes mainly involved in chemokine-mediated, and complement receptor-mediated signaling pathways; and response to interferon-gamma, lipopolysaccharide, interferon-gamma, and chemokines. The cellular component (CC) was bent on the plasma, lysosomal, lytic vacuole membrane. Molecular function (MF) mainly enriched on the chemokine activity (Additional file 12: Table S2).

### Identification and validation of hub genes

**Fig. 5:**
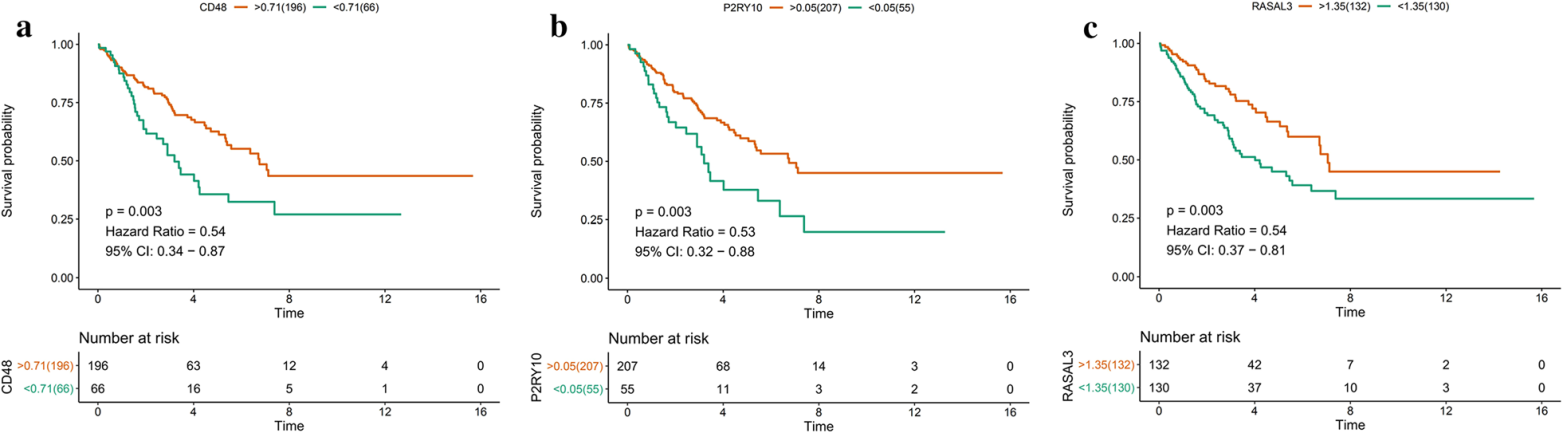
Overall survival in relation to the three genes. **a** CD48; **b** P2RY10; **c **RASAL3

Based on the following criteria (|MM|> 0.8, |GS|> 0.2, and the largest sub-network), four genes with high connectivity in the clinically significant modules were identified as hub genes (Additional file 13: Figure S11). The “survival” package in R was performed to calculate the survival analysis (*p* < 0.05 as statistically significant). Table 2 shows that the survival analysis of hub genes identified CD48 antigen (*CD48*), putative P2Y purinoceptor 10 (*P2RY10*), and RAS protein activator like-3 (*RASAL3*). Figure 5 shows that these three genes were significantly and positively associated with overall survival. In addition, immunohistochemistry (IHC) staining data acquired from the HPAD also confirmed the differential expression of the predicted genes (*CD48, P2RY10, RASAL3*) in sarcoma samples (Additional file 14: Figure S12).

**Table 2:** Hub genes in the significant module

Gene symbol	Co-expression analysis		MCODE analysis	
	GS	MM	Connectivity degree	MCODE_score
**CD48**	0.51	0.95	24	18
**P2RY10**	0.50	0.89	17	16.83
**RASAL3**	0.58	0.95	23	16.83

### Genetic alterations

**Fig. 6:**
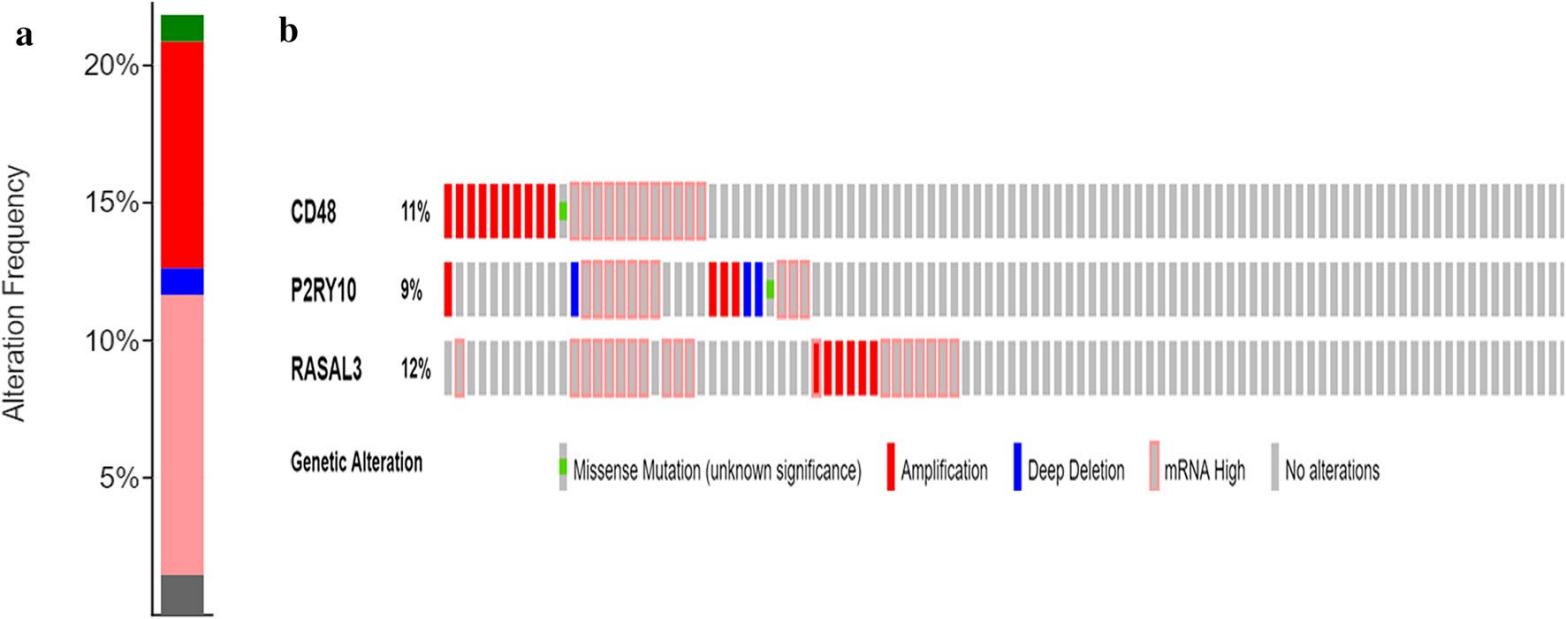
Genetic alterations. **a** Alteration frequency of each of the three hub genes in sarcoma tumors; **b** Visual summary of genetic alternations in the three hub genes

Analyzing multi-dimensional cancer genomics changes and clinical data. In this study, we used the CBioPortal database to estimate the genetic alterations in *CD48*, *P2RY10*, and *RASAL3*. Fig. 6a shows that the frequency of mutations in *CD48* was 11% in *P2RY10* it was 9%, and in *RASAL3* it was 12%. The three genes were altered in 22% (45/206) of the patients (Fig. 6b).

## Discussion

Sarcomas, originating from mesenchymal cells, are comprehensive tumors with poor prognoses. There are no obviously clinical symptoms at the early stage of the disease, and distant metastasis occurs at the late stage. However, its potential molecular mechanism is still unclear. Among the available therapeutic strategies, immunotherapy is considered the safest and most effective. In this study, tumor-infiltrating immune cells and the tumor-microenvironmental scores were used to construct a co-expression network to identify genes associated with the prognosis and metastasis of sarcomas (Fig. 1).

We used the CIBERSORT algorithm to identify different patterns of tumor-infiltrating lymphocytes in sarcoma samples, and characterize corresponding clinical traits. TILs are an indicator of tumor inflammation infiltration, and it has been shown that TIL subsets perform essential functions in the development of malignancies. In the sarcoma tumors, 35–40% of the TILs were macrophages. Among the various TIL parameters, the macrophages were the predominant immune cell type in sarcoma tissues. Macrophages are myeloid immune cells that are strategically positioned throughout body tissue and they can present antigens on their external membrane, which can promote the release of inflammatory cytokines by sarcoma tumors [38]. Macrophages are major components of the tumor microenvironment and orchestrate various aspects of the immune response to sarcomas. Cells differentiate into uncommitted macrophages (M0), and subsequently activated into pro-(M1) and anti-inflammatory (M2) phenotypes, which affect tumorigenesis by either promoting cytokine release by immune cells or by enhancing the antitumor response [39, 40]. Of these subtypes, M1 macrophages involved in inflammation and anti-tumor response [41], and M2 macrophages promote tumor growth in sarcoma. The accumulated immune regulatory cells from peripheral monocytes in the tumor microenvironment (TME) not only prevents T lymphocytes from attacking the tumor but also secrete cytokines to nourish, leading tumor metastasis [42]. The Kaplan–Meier analysis suggests that macrophages are associated with disease type, overall survival, and percent necrosis in sarcoma tumors (Figs. 2, 3). In addition, our results shown that the fraction of CD8 T cells was negatively associated with M0 macrophages (R = −0.43) and M2 macrophages (R = −0.41), and positively associated with M1 macrophages (R = 0.51) and follicular, helper T cells (R = 0.6)(Fig. 2c). As previous reported, in various cancer types, the numbers and activation states of immune effectors cells, in particular, CD8 T cells, are the primarily cell type responsible for immune therapies responses, as they could specifically recognize and kill cancer cells by secreting cytokines and cytotoxic molecules [43]. Among sarcomas subtypes, CD8 T cell immunity varies significantly and may better explain the varied clinical effects of immunotherapies [44]. Previous report showed that TGF-β suppresses CD8+ effector T-cell function, inhibits the Th1 phenotype, and activates M2 macrophages polarization, driving immune cells from the tumor compartment [45]. We found that the CD8 T cell fraction was negatively associated with M0 and M2 macrophages, and positively associated with M1 macrophages in sarcomas samples, consistent with the previous reports [46, 47], suggesting that CD8 T cells and macrophages may be potential markers for the prognosis of patients with sarcoma.

TME is a complex, integrated system, which is different from the microenvironment established by normal cells and their surrounding tissue [48]. The TME plays a pivotal role in tumor progression and metastasis, and may significantly influence therapeutic response to cancer treatment [49, 50]. In this study, we calculated the Immune-, Stromal- and Estimate- scores for sarcoma samples by applying the ESTIMATE algorithm. As shown in Fig. 4 and Table 1, disease type and gender significantly increased with higher Estimate, Immune, and Stromal scores. These data suggest that the high scoring-groups have a better prognosis. Then, genes differentially expressed between low- and high- immune/stromal score groups were identified and characterized as having more or less DEGs.

WGCNA is a systematic biological algorithm [26], which is used to reveal the association between genes and clinical phenotypes [51]. It has been widely used to identified potential biomarkers for Alzheimer’s disease [52], breast cancer [27], and osteoarthritis [53]. In this paper, we identified 7 modules via WGCNA (Additional files 6,7, 9: Figures S6-S8). Among these, the blue module was significantly associated with immune cell subtypes related to CD8 T cell. KEGG analysis revealed that the genes in blue module were mainly enriched for cytokine/cytokine receptor interaction, chemokine signaling pathways, the PI3K-AKT signaling pathway, and the JAK-STAT signaling pathway (Additional file 11: Figure S10). Proinflammatory cytokines are involved in cancer progression, and cytokine/cytokine receptor interactions may be essential mediators of inflammation in the development and prognosis of sarcomas [48]. Our data indicate that the cytokine/cytokine receptor signaling pathway is involved in sarcoma progression [54]. Chemokine signaling helps coordinate cell migration [55]. The JAK-STAT signaling pathway transfer signals from cell membrane receptors to the nucleus in sarcoma tumors [56]. It modulates the activity of immune system, especially the fate of helper T cells [57]. Zhang et al. found that downregulated expression of HGDF promotes tumor development and progression by coordinating the PI3K-AKT signaling pathway in sarcomas. In addition, GO analysis has shown that the blue module is predominantly involved in chemokine-mediated and complement receptor-mediated signaling and chemokine activity. Previous studies have confirmed that the plasma, lysosomal, and lytic vacuole membranes may be potential targets for treating sarcomas [58–61].

In the blue module, *CD48*, *P2RY10*, and *RASAL3* were identified as differentially expressed (Figs. 5, 6, and Table 2). They were associated with survival analysis results and were validated at the protein level. *CD48*, a member of CD2 immunoglobulin superfamily (IgSF) participates in activation and differentiation pathways in CD84, CD150, CD229 and CD244 [62]. Liu et al*.* [63] suggested CD48 as a key gene for the induction of histiocytic sarcoma in mouse skeletal muscle. *P2RY10* belongs to the family of G -protein -coupled receptors, which are activated by adenosine and uridine [64]. Wang et al. found that P2RY10 was potentially involved in the immune response and the development of sarcomas [65]. *RASL3*, a novel member of the RasGAP Rasal family, is predominantly expressed by T cells [66, 67], and RasGAP activity stimulates ERK phosphorylation. Previous studies have not linked RASL3 to the cancer [68]; therefore, it may be a novel immunotherapy target for the treatment of patients with sarcoma.

## Conclusions

In this study, we demonstrated novel insights into immune infiltration and immune microenvironment of sarcomas. CD8 T cell and macrophages infiltration revealed important associations with sarcomas, and immune scores significantly correlated with sarcomas. Three hub genes (*CD48, P2RY10, and RASAL3*) associated with immunotherapy and the development of sarcomas were analyzed and presented, as potential prognostic biomarkers and/or therapeutic targets of immunotherapy for sarcomas.

## Supplementary Information

**Additional file 1: Figure S1.** Heatmap of immune cells estimated.

**Additional file 2: Figure S2.** The fraction of (A) activated dendritic cells, (B) resting dendritic cells, (C) M2 macrophages, (D) neutrophils, and (E) plasma cells by disease type. The fraction of (F) activated dendritic cells and (G) M2 macrophages among disease recurrence. The fraction of (H) activated dendritic cells, (I) resting NK cells, and (J) follicular helper T cells among total necrosis percent.

**Additional file 3: Figure S3.** The relationship between Estimate-, Immune-, and Stromal scores of sarcoma tumors and clinical parameters of sarcoma patients. (A) Estimate scores and age; (B) Estimate scores and disease type (*p* < 0.05); (C) Estimate scores and gender (*p* < 0.05); (D) Estimate scores and disease recurrence; (E) Estimate scores and percent of necrosis; (F) Immune scores and age (*p* < 0.05); (G) Immune scores and disease type (*p* < 0.05); (H) Immune scores and gender (*p* < 0.05); (I) Immune scores and disease recurrence; (J) Immune scores and percent of necrosis; (K) Stromal scores and age; (L) Stromal scores and disease type (*p* < 0.05); (M) Stromal scores and gender (*p* < 0.05); (N) Stromal scores and disease recurrence; (O) Stromal scores and percent of necrosis.

**Additional file 4: Figure S4.** Heatmap of DEGs in the groups with low- and high- scores. (A) Immune scores; (B) Stromal scores.

**Additional file 5: Figure S5.** Common DEGs in immune- and stromal- scores. (A) Commonly up-regulated genes; (B) Commonly down-regulated genes.

**Additional file 6: Figure S6.** (A) Hierarchical clustering dendrogram of samples; (B) Co-expression network modules. (C) Median rank and Zsummary statistics of the most variant gene module preservation.

**Additional file 7: Figure S7.** (A) The scale-free fit index for soft-thresholding powers; (B) The mean connectivity for soft-thresholding powers.

**Additional file 8: Table S1.** The Zsummary.pres and Zsummary.qual of modules.

**Additional file 9: Figure S8.** (A) Heatmap of the correlation between module eigengenes and immune cell types; (B) Network dendrogram and heatmap between the module and immune types; (C) Interaction of co-expressed genes; (D) Multidimensional scaling (MDS) plots representing the co-expression network.

**Additional file 10: Figure S9.**Multi-dimensional scaling (MDS) plot of the co-expression network.

**Additional file 11: Figure S10.** Pathway enrichment and GO analysis of the blue module. (A) KEGG pathway analysis; (B) Biological process (BP) analysis; (C) Cellular component (CC) analysis; (D) Molecular function (MF) analysis.

**Additional file 12: Table S2.** The GO enrichment and KEGG pathway enrichment in the blue module.

**Additional file 13: Figure S11.** Gene identification. (A) Scatter plot of eigengenes in the blue module; (B) PPI network of genes in the MCODE sub-network.

**Additional file 14: Figure S12.** Immunohistochemical analysis of (A) CD48, (B) P2RY10, (C) RASAL3 expression.

## Data Availability

Not applicable.

## Abbreviations

BP: Biological process
CC: Cellular component
CD48: CD48 antigen
CIBERSORT: Cell-Type Identification by Estimating Relative Subsets of RNA Transcripts
DEGs: Differentially expressed genes
FC: Fold change
FDR: False discovery rate
GDC: Genomic Data Commons
GO: Gene ontology
GS: Gene significance
HPAD: Human Protein Atlas Database
IHC: Immunohistochemistry
KEGG: Kyoto Encyclopedia of Genes and Genomes
MDS: Multidimensional scaling
Mes: Module eigengenes
MF: Molecular function
MS: Module significance
PPI: Protein–protein interaction
P2RY10: Putative P2Y purinoceptor 10
RASAL3: RAS protein activator like-3
TCGA: The Cancer Genome Atlas
TIL: Tumor-infiltrating lymphocytes
TME: Tumor microenvironment
TOM: Topological matrix
WGCNA: Weighted gene co-expression network analysis
