# Supplementary figures and images for: Assessing immune infiltration and the tumor microenvironment for the diagnosis and prognosis of sarcoma
Source: Cancer Cell Int. 2020 Dec 2;20:577. doi: 10.1186/s12935-020-01672-3 (PMC7709254; doi:10.1186/s12935-020-01672-3)

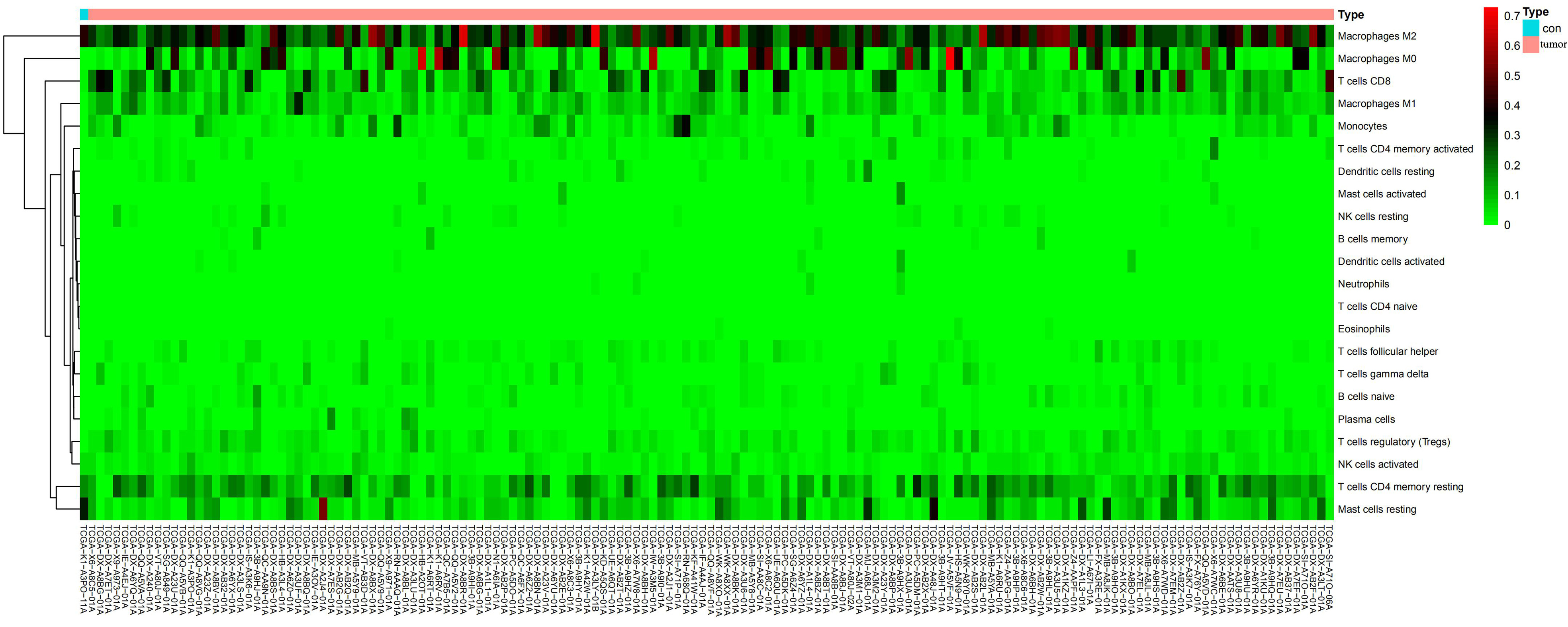

Supplement: Supplementary file 1 — Additional file 1: Figure S1. Heatmap of immune cells estimated. [file 12935_2020_1672_MOESM1_ESM.jpg]

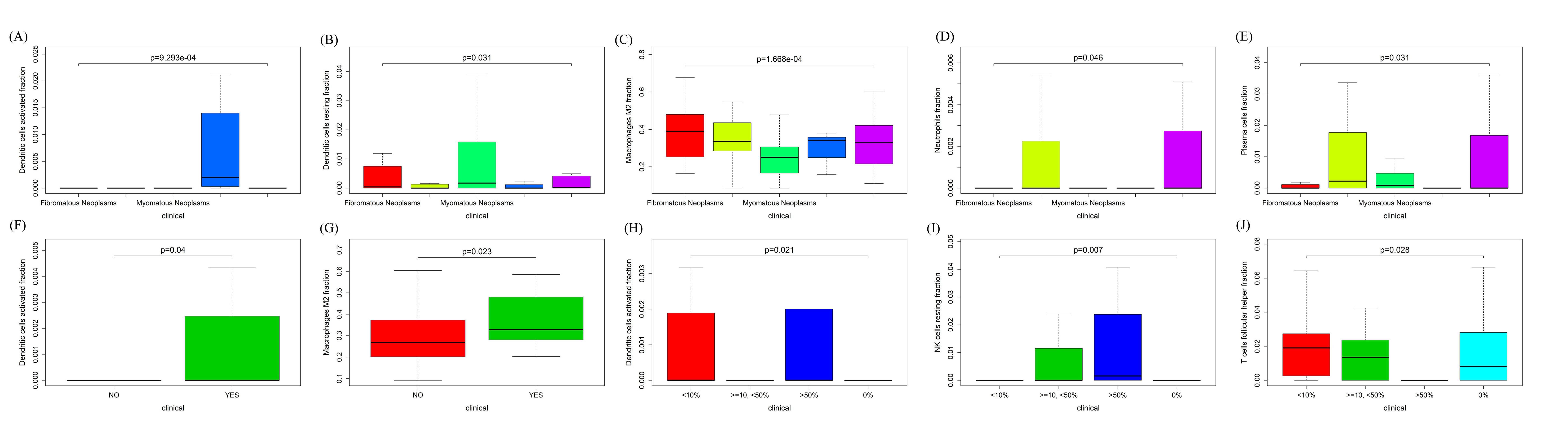

Supplement: Supplementary file 2 — Additional file 2: Figure S2. The fraction of (A) activated dendritic cells, (B) resting dendritic cells, (C) M2 macrophages, (D) neutrophils, and (E) plasma cells by disease type. The fraction of (F) activated dendritic cells and (G) M2 macrophages among disease recurrence. The fraction of (H) activated dendritic cells, (I) resting NK cells, and (J) follicular helper T cells among total necrosis percent. [file 12935_2020_1672_MOESM2_ESM.jpg]

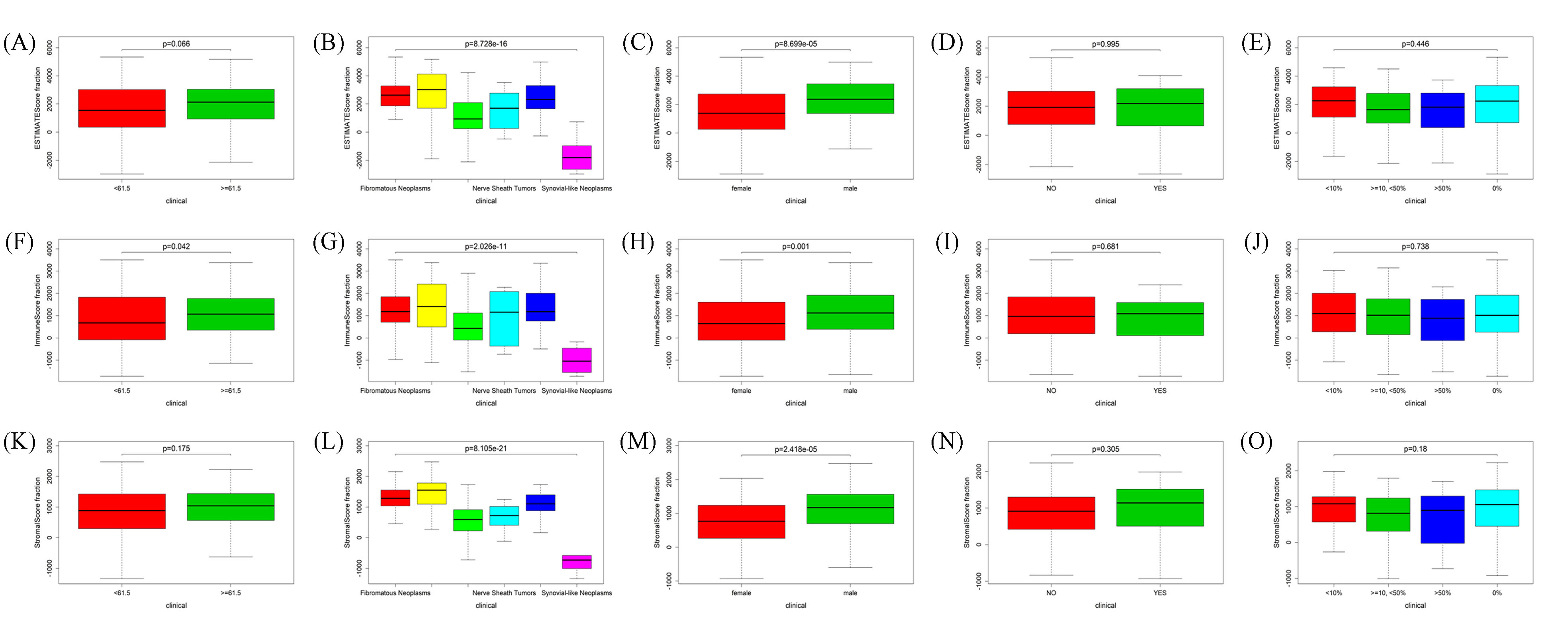

Supplement: Supplementary file 3 — Additional file 3: Figure S3. The relationship between Estimate-, Immune-, and Stromal scores of sarcoma tumors and clinical parameters of sarcoma patients. (A) Estimate scores and age; (B) Estimate scores and disease type (p < 0.05); (C) Estimate scores and gender (p < 0.05); (D) Estimate scores and disease recurrence; (E) Estimate scores and percent of necrosis; (F) Immune scores and age (p < 0.05); (G) Immune scores and disease type (p < 0.05); (H) Immune scores and gender (p < 0.05); (I) Immune scores and disease recurrence; (J) Immune scores and percent of necrosis; (K) Stromal scores and age; (L) Stromal scores and disease type (p < 0.05); (M) Stromal scores and gender (p < 0.05); (N) Stromal scores and disease recurrence; (O) Stromal scores and percent of necrosis. [file 12935_2020_1672_MOESM3_ESM.jpg]

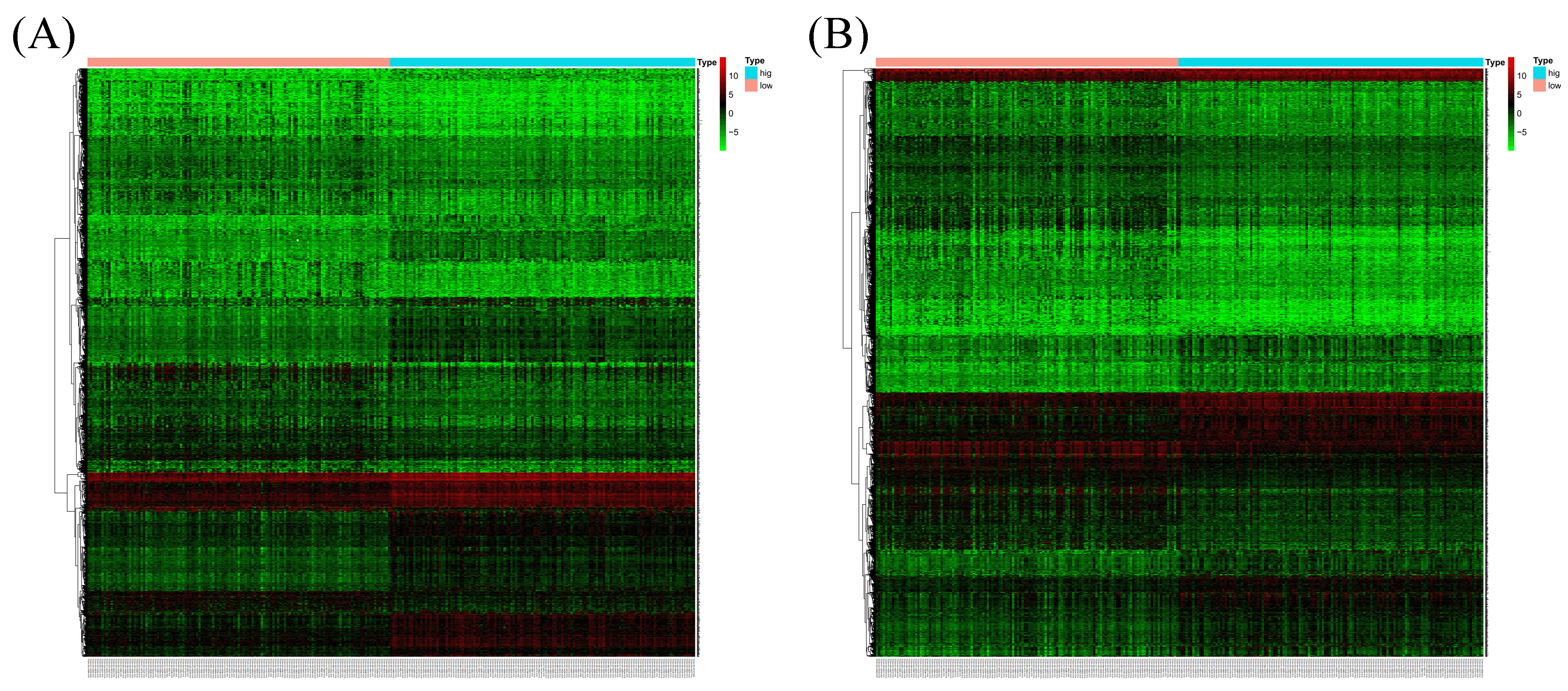

Supplement: Supplementary file 4 — Additional file 4: Figure S4. Heatmap of DEGs in the groups with low- and high- scores. (A) Immune scores; (B) Stromal scores. [file 12935_2020_1672_MOESM4_ESM.jpg]

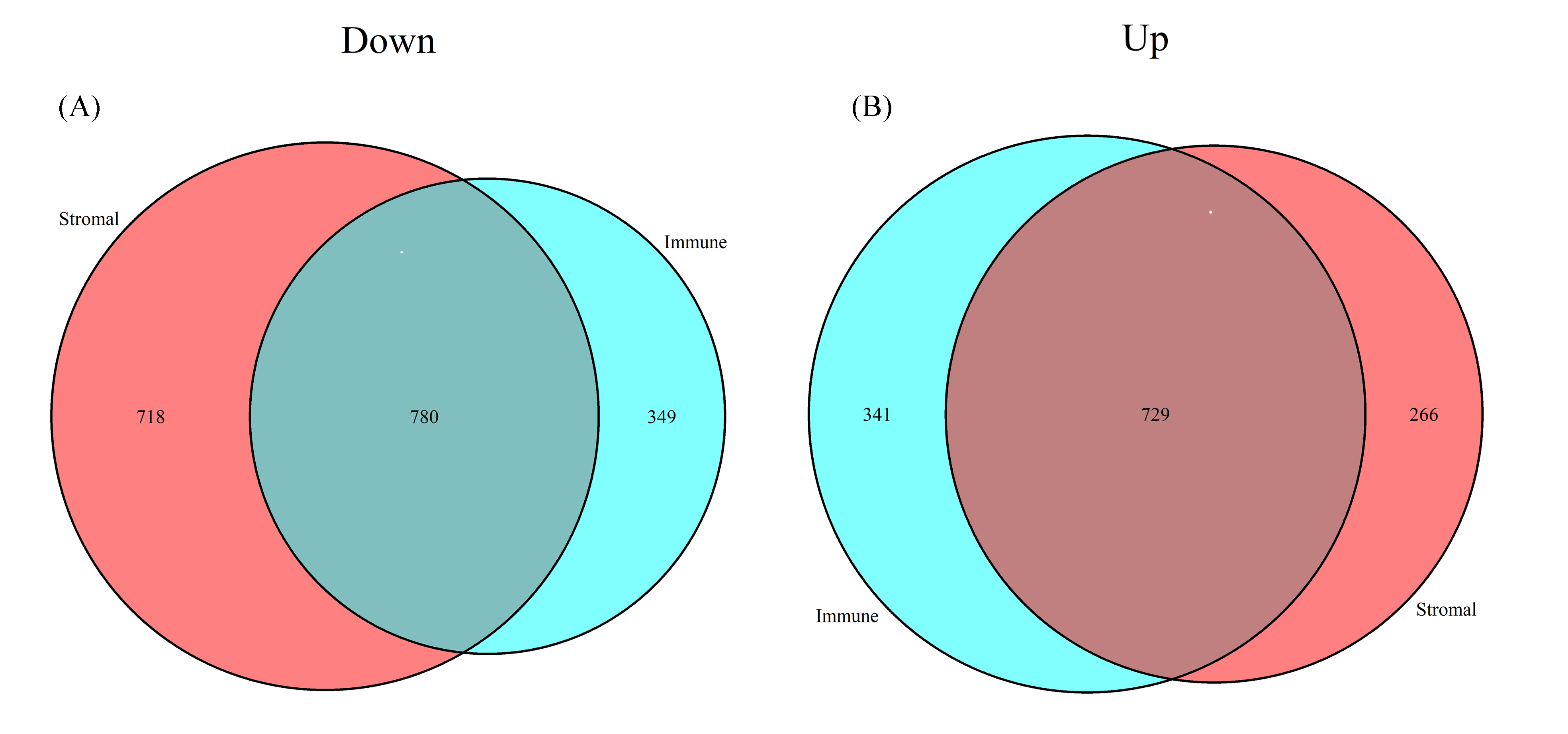

Supplement: Supplementary file 5 — Additional file 5: Figure S5. Common DEGs in immune- and stromal- scores. (A) Commonly up-regulated genes; (B) Commonly down-regulated genes. [file 12935_2020_1672_MOESM5_ESM.jpg]

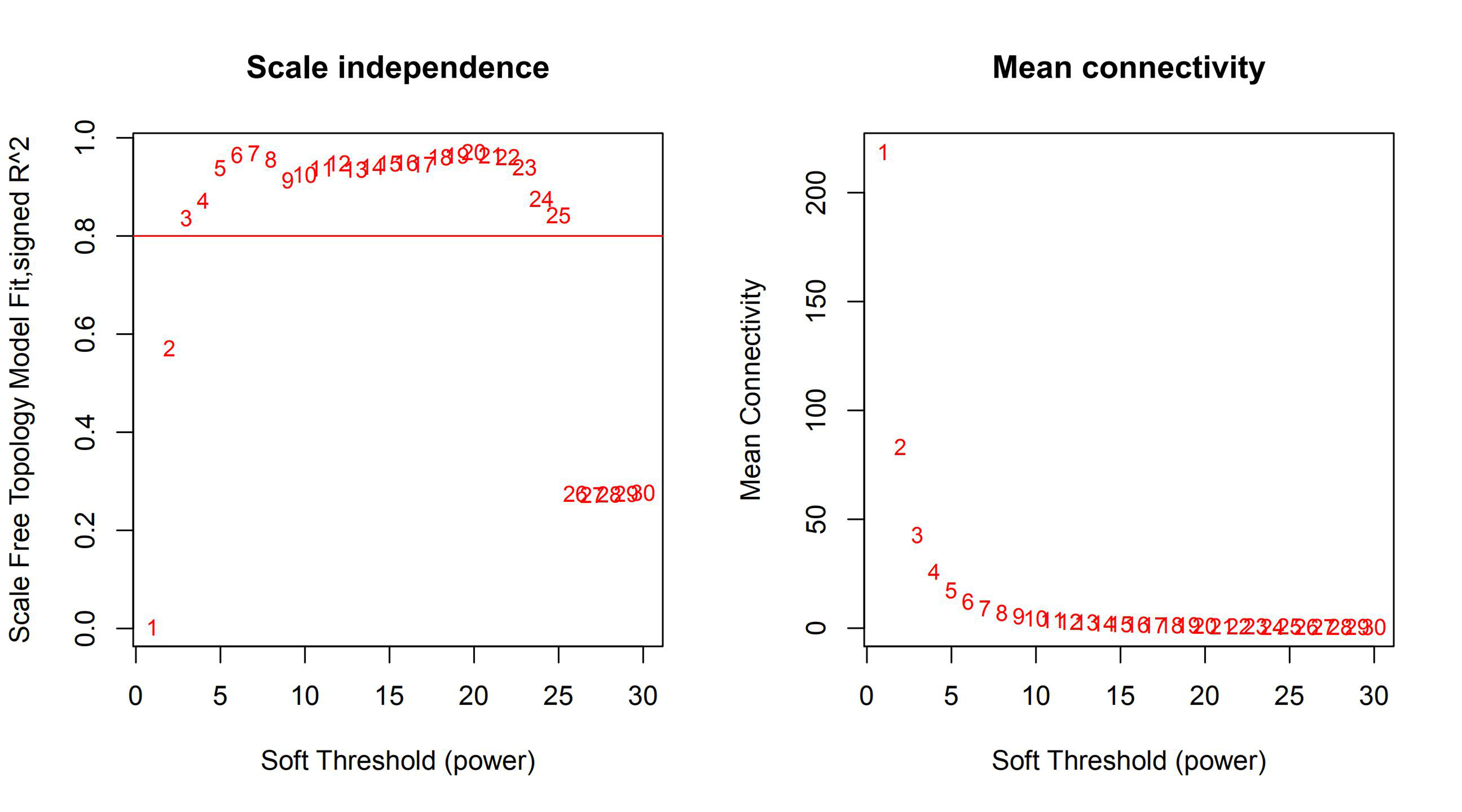

Supplement: Supplementary file 7 — Additional file 7: Figure S7. (A) The scale-free fit index for soft-thresholding powers; (B) The mean connectivity for soft-thresholding powers. [file 12935_2020_1672_MOESM7_ESM.jpg]

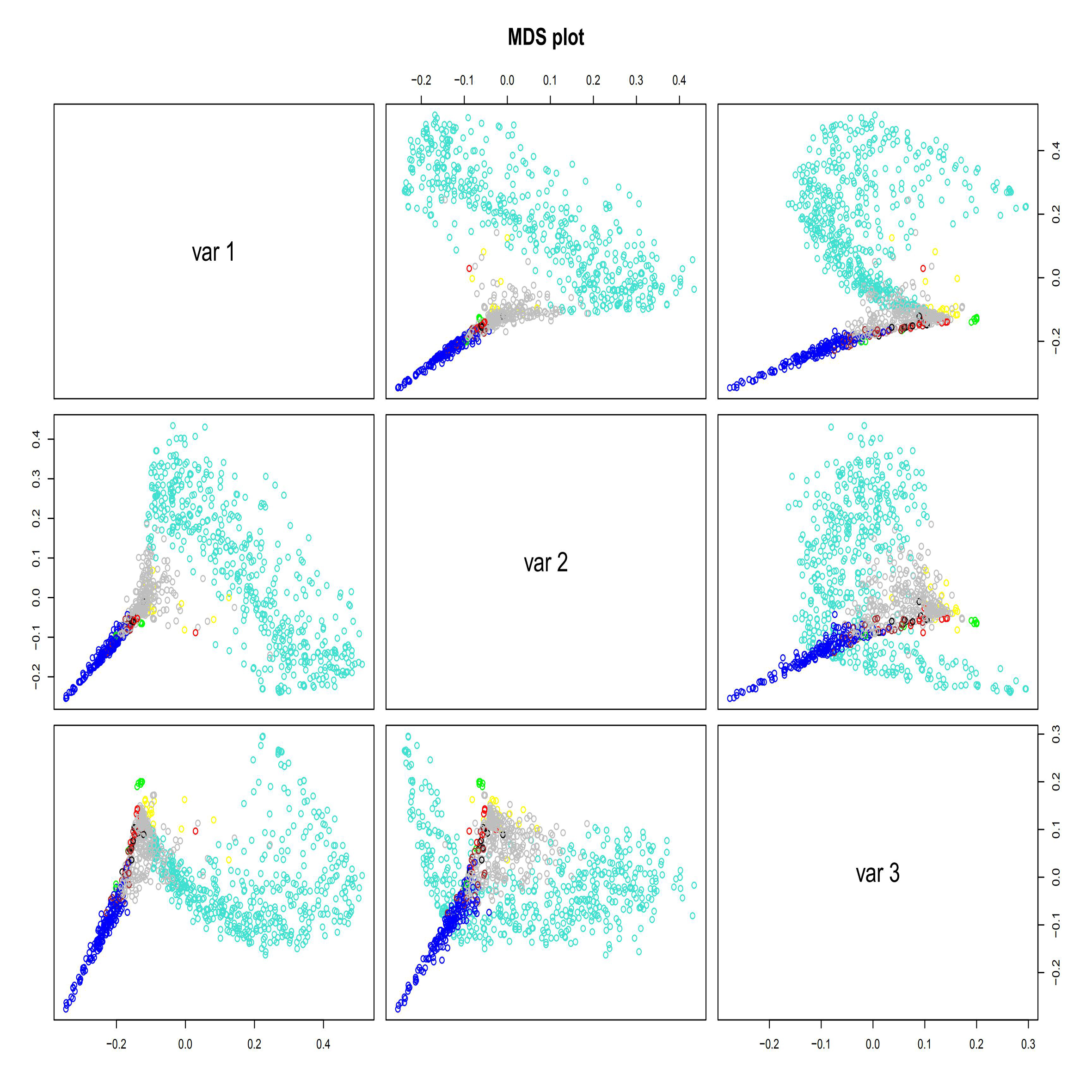

Supplement: Supplementary file 10 — Additional file 10: Figure S9.Multi-dimensional scaling (MDS) plot of the co-expression network. [file 12935_2020_1672_MOESM10_ESM.jpg]

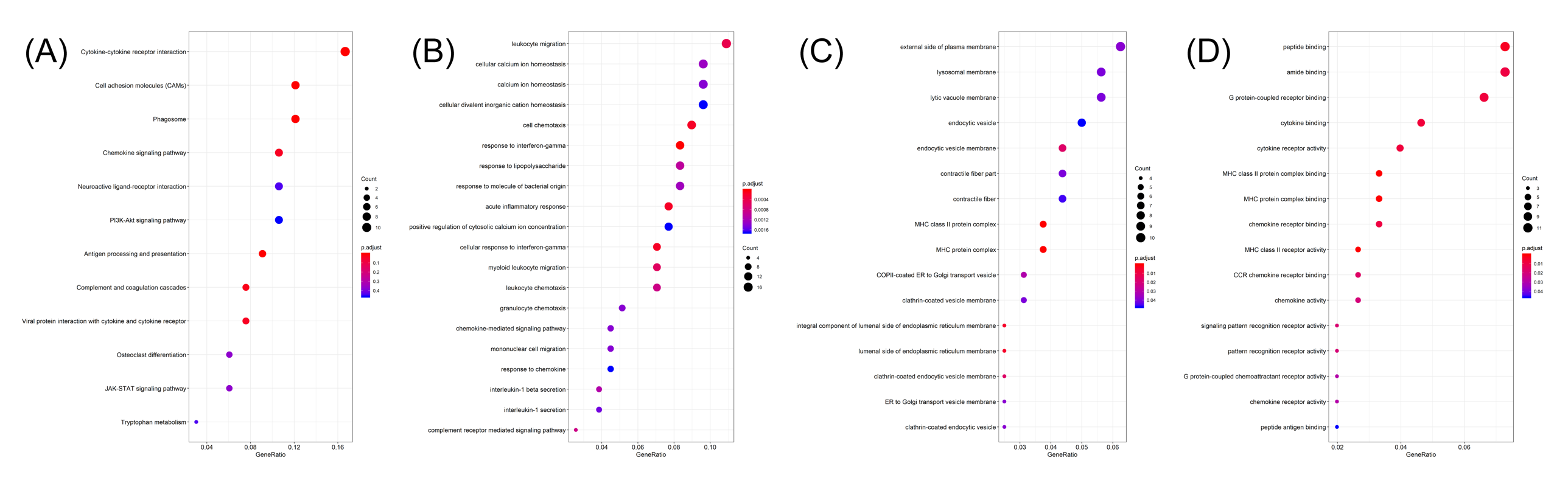

Supplement: Supplementary file 11 — Additional file 11: Figure S10. Pathway enrichment and GO analysis of the blue module. (A) KEGG pathway analysis; (B) Biological process (BP) analysis; (C) Cellular component (CC) analysis; (D) Molecular function (MF) analysis. [file 12935_2020_1672_MOESM11_ESM.jpg]

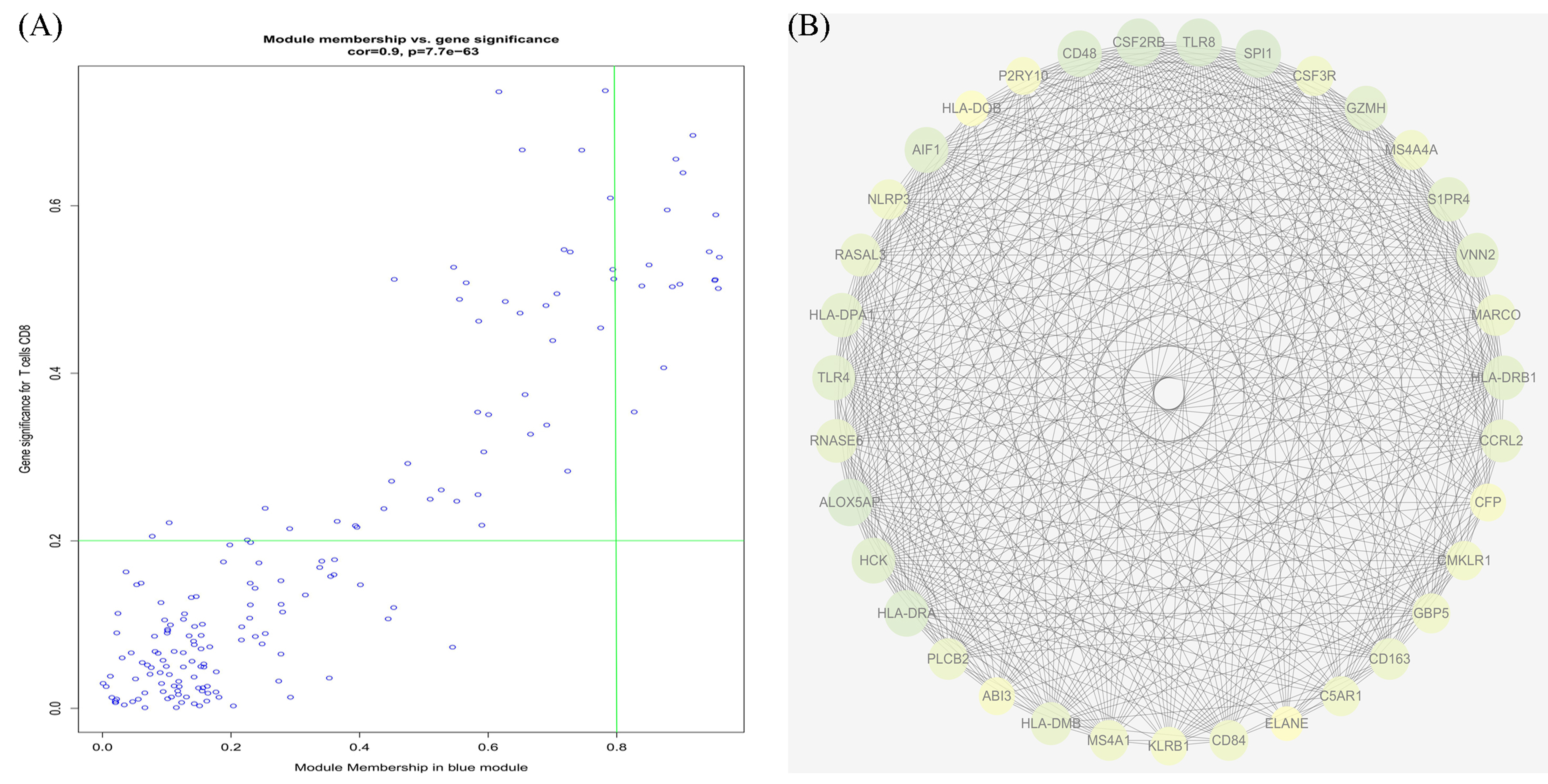

Supplement: Supplementary file 13 — Additional file 13: Figure S11. Gene identification. (A) Scatter plot of eigengenes in the blue module; (B) PPI network of genes in the MCODE sub-network. [file 12935_2020_1672_MOESM13_ESM.jpg]

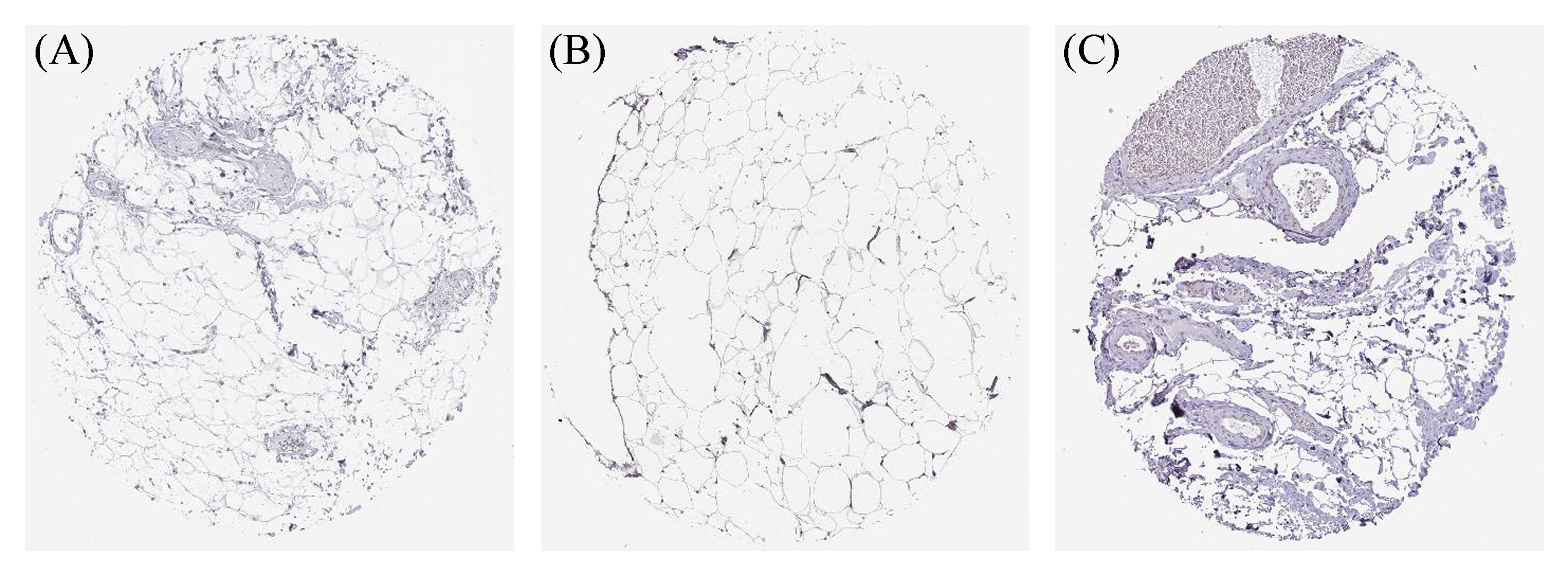

Supplement: Supplementary file 14 — Additional file 14: Figure S12. Immunohistochemical analysis of (A) CD48, (B) P2RY10, (C) RASAL3 expression. [file 12935_2020_1672_MOESM14_ESM.jpg]
